# Hydrodynamic Radii of Intrinsically Disordered Proteins: Fast Prediction by Minimum Dissipation Approximation and Experimental Validation

**DOI:** 10.1021/acs.jpclett.4c00312

**Published:** 2024-05-02

**Authors:** Radost Waszkiewicz, Agnieszka Michaś, Michał K. Białobrzewski, Barbara P. Klepka, Maja K. Cieplak-Rotowska, Zuzanna Staszałek, Bogdan Cichocki, Maciej Lisicki, Piotr Szymczak, Anna Niedzwiecka

**Affiliations:** † Institute of Theoretical Physics, Faculty of Physics, University of Warsaw, L. Pasteura 5, 02-093 Warsaw, Poland; ‡ Institute of Physics, Polish Academy of Sciences, Aleja Lotnikow 32/46, PL-02668 Warsaw, Poland

## Abstract

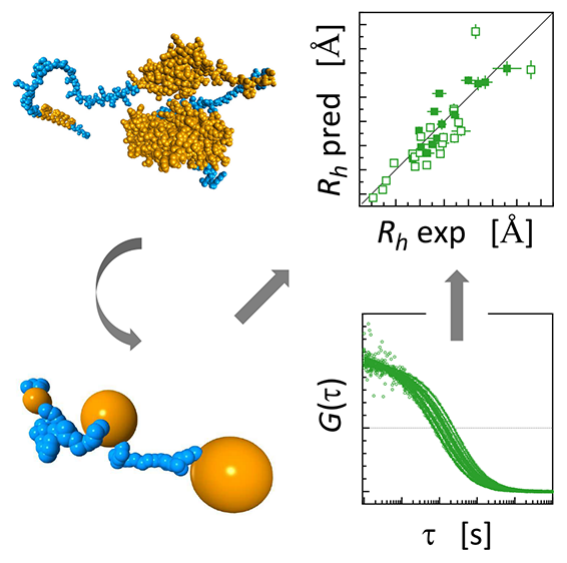

The diffusion coefficients of globular and fully unfolded proteins can be predicted with high accuracy solely from their mass or chain length. However, this approach fails for intrinsically disordered proteins (IDPs) containing structural domains. We propose a rapid predictive methodology for estimating the diffusion coefficients of IDPs. The methodology uses accelerated conformational sampling based on self-avoiding random walks and includes hydrodynamic interactions between coarse-grained protein subunits, modeled using the generalized Rotne−Prager−Yamakawa approximation. To estimate the hydrodynamic radius, we rely on the minimum dissipation approximation recently introduced by Cichocki et al. Using a large set of experimentally measured hydrodynamic radii of IDPs over a wide range of chain lengths and domain contributions, we demonstrate that our predictions are more accurate than the Kirkwood approximation and phenomenological approaches. Our technique may prove to be valuable in predicting the hydrodynamic properties of both fully unstructured and multidomain disordered proteins.

Intrinsically disordered proteins (IDPs) constitute an extensive class of biological macromolecules, and their role in the homeostasis of a living cell has been increasingly recognized in recent decades.^1,2^ The frequency of long intrinsically disordered regions (IDRs) in proteins differs significantly between the kingdoms of life, ranging from 2% in archaea to 33% in eukaryotes.^3^ The IDP molecules display different degrees of structural disorder. Their chains can encompass either several folded globular domains or supersecondary structures connected by flexible linkers, sparse secondary structural elements, or can be completely natively unstructured. Disordered proteins exhibit a notable characteristic, the absence of a stable, well-defined relative spatial arrangement of their fragments. Instead, their equilibrium properties can be described through a broad set of rapidly interconverting conformers, posing a challenge for analysis, particularly in the context of long chains.^4^

The average geometric properties of IDPs, including their shape and size, are determined by the equilibrium ensemble of conformational states. This equilibrium state is intricately influenced by environmental conditions,^5^ such as temperature,^6^ ionic strength,^7,8^ osmolality,^9^ crowding,^10^ post-translational modifications,^11^ and the presence of specific molecular binding partners.^12^ The formation of transient or more stable noncovalent complexes introduces another nontrivial dependence of the IDP equilibrium geometry on environmental factors.

Because the shape and availability of the binding sites necessary for the interaction of IDP with ligands, other proteins, and nucleic acids are strongly influenced by the environment, IDPs often act as higher-order regulators in key cellular processes such as gene expression,^11,13^ signaling,^2,14^ or extracellular biomineralization.^15^ The different conformations of these flexible proteins enable IDPs to perform their multiple functions.^1^ In particular, it is worth emphasizing the important roles of IDPs in health and disease, e.g., the role of the p53 protein as a tumor suppressor,^16^ mutations of which are often responsible for human cancers, the function of 4E-BPs in the inhibition of eukaryotic translation initiation,^11,17−19^ the significance of GW182 protein in the recruitment of the multiprotein machinery necessary for microRNA-mediated gene silencing,^20−22^ or the importance of Tau, FUS, and α-synuclein proteins in neurodegenerative diseases.^23,24^ Because the elastic properties of these biomolecules are responsible for the proper functioning of IDPs in the cellular context, i.e., for the association of complexes and the formation of biomolecular condensates via liquid–liquid phase separation such as, e.g., RNA-processing membraneless organelles,^25,26^ much attention has been paid to the hydrodynamic properties of IDPs. Experimental techniques, such as analytical ultracentrifugation (AUC), size exclusion chromatography (SEC), pulsed-field gradient nuclear magnetic resonance (PFG-NMR), dynamic light scattering (DLS), and fluorescence correlation spectroscopy (FCS), offer insights into hydrodynamic parameters (as reviewed by Białobrzewski et al.^27^). However, due to the distinct limitations of each experimental approach, ongoing research aims to devise phenomenological methods for calculating the hydrodynamic radius (*R*_h_). These methods may involve deriving *R*_h_ from the radius of gyration (*R*_g_) determined by small-angle X-ray scattering (SAXS)^28,29^ or exploiting the conformational backbone propensity of IDPs.^30,31^ However, it has recently been noted that inferring structural properties of the IDP conformational ensembles from SAXS is prone to a high degree of uncertainty.^32^

A theoretical Monte Carlo approach was also developed on the basis of a bead chain model showing that proper consideration of the excluded volume effect is critical for estimating the *R*_h_ value of the disordered N-terminal Sic1 fragment,^33^ in accordance with FCS experimental results.^34^

Simultaneously, significant effort is being invested in developing numerical models that extract the characteristics of IDPs from conformational ensembles obtained using molecular dynamics (MD) simulations, deep learning, or energy minimization algorithms.^35−47^ However, the molecular flexibility of IDPs introduces substantial complexities when determining their hydrodynamic properties. Two main issues here are the large number of degrees of freedom and the long time scales of relaxation of the internal coordinates of the molecules. These factors prohibit direct calculation of the experimentally relevant long-time diffusion coefficient from either molecular or Brownian dynamics trajectories. One popular approximation that circumvents this difficulty is to assume that the macromolecule is rigidly frozen in one of a large number of possible conformations. Transport properties are then calculated by treating the molecule as a rigid body, and the results are averaged over an equilibrium ensemble.^48−51^ Nevertheless, the validity and accuracy of this approximation remain uncertain. Additionally, the generation of conformational ensembles can be a bottleneck for long chains (beyond ∼300 amino acid residues) because it requires time-consuming MD simulations and/or the construction of new databases of short peptide conformations.

There is, therefore, a strong need to develop a numerically efficient solution that would enable reliable calculation of the long-time diffusion coefficient of any long chain IDP, such as one with 1000 amino acid residues, solely on the basis of its sequence information.

In this study, we introduce a new theoretical approach for both generating conformational ensembles of IDPs and calculating their hydrodynamic properties. This method enables a swift estimation of the diffusion coefficient for long IDPs in a matter of minutes, with superior accuracy compared to that of existing methods. This assertion is substantiated through rigorous testing of the model on a diverse set of experimental results obtained for 43 proteins. The data set includes both literature data and *R*_h_ values measured for a set of new IDP constructs using FCS under mild conditions (see the Supporting Information).

We present our results in terms of the hydrodynamic radius of a molecule, *R*_h_. This radius represents the size of a solid sphere that possesses the same translational diffusion coefficient, *D*, as the given molecule under identical buffer conditions. Therefore, *R*_h_ = *k*_B_*T*/6*πηD*, where *T* is the temperature and η is the viscosity.

An important observation by Fixman^52,53^ is that the diffusion coefficient of a flexible macromolecule is time-dependent, with well-defined short- and long-time limits. The disparity between the two is attributed to the effects associated with relaxation of the internal coordinates of the molecule, as well as rotation of the macromolecule as a whole.^52,54,55^ The positivity of the dissipation rate in the system implies that the long-time diffusion coefficient (*D*_l_) is always smaller than the short-time diffusivity (*D*_s_).^53^ The focus of theoretical approaches should be the determination of the former quantity, as it is the one measured in experiments utilizing techniques like FCS, AUC, or DLS. Unfortunately, the calculation of *D*_l_ is significantly more challenging than that of *D*_s_ because it involves the computation of time-dependent quantities, such as the memory function, which describes the relaxation effects. An additional point to keep in mind is that the value of the short-time diffusion coefficient depends on the choice of the point that one tracks.^55−58^ In contrast, the long-time diffusivity is independent of the choice of reference point.^59^

The methods for predicting the diffusion coefficient can be broadly split into three categories: atomistic, phenomenological, and coarse-grained. For small proteins, high-resolution, atomistic MD methods can be used,^60^ but they require either simulating the surrounding water molecules explicitly, which is very computationally intensive, or an implicit solvent scheme. In the case of implicit solvent methods, addressing hydrodynamic interactions between distant parts of the molecule^61−64^ and thermalization^65^ pose significant challenges. Additionally, even for the smallest proteins, it is prohibitively difficult to obtain statistically meaningful data over the 10–100 ms scale, which would enable the direct computation of the long-time diffusion coefficient.

The other extreme consists of phenomenological models that predict *R*_h_ from the number of residues *N* and possibly other parameters, such as the total charge or amino acid composition. Theoretical considerations of Rouse, who modeled a protein as a Gaussian chain,^66^ provided a foundation to the power law relationship *R*_h_ ∼ *N*^1/2^. The classical Rouse model employs random displacements between the monomers. If we assume complete independence of displacements between each consecutive pair of monomers, the central limit theorem dictates that as *N* approaches infinity, the squared end-to-end distance should conform to a scaled χ^2^(3) distribution. Consequently, the dimensions of such an idealized chain are expected to scale with . Later work of Zimm included the effect of excluded volume,^67^ which resulted in the scaling *R*_h_ ∼ *N*^γ^ with γ = 0.588.

Phenomenological size–length relationships that include other variables involve a number of fitting parameters. As a result, their range of applicability outside of the fitting data set is difficult to assess. An alternative phenomenological approach proposed by Pesce et al.^29^ employs the radius of gyration obtained from SAXS experiments to estimate *R*_h_. This is substantiated by the observation that within the Kirkwood–Riseman approximation^68^*R*_h_ and *R*_g_ share the same scaling relationship with *N* as long as the pair-displacement distribution converges under appropriate scaling to a Gaussian for large *N* values.

Finally, coarse-grained models, like our method, employ larger units (typically one or two per amino acid residue) as building blocks for the structure prediction scheme, along with approximate interaction potentials between subunits, to simulate the equilibrium ensemble of configurations for a given molecule. These configurations are then combined with an approximation of the hydrodynamic properties to compute the diffusion coefficient. Essentially, the computation of the latter for elastic macromolecules addresses two interconnected challenges: predicting the conformations of molecules on the basis of available biochemical data and then using these conformations to predict hydrodynamic properties.

The different exponents in the power law relationships of Rouse^66^ and Zimm^67^ demonstrate that even the most basic method for approximating configurations must take into account excluded volume interactions.

A software that can accommodate excluded volume interactions for a disordered chain is Flexible Meccano (FM).^37^ In addition to volume exclusion, it considers the distribution of Ramachandran angles determined from crystallographic protein structures when sampling conformations. However, FM treats the entire chain as unstructured, so it cannot be used to model proteins that possess both globular and unfolded segments, which are in fact much more common than fully unstructured chains. Unfortunately, FM has a closed license that precludes necessary modifications to accommodate folded regions of proteins.

The complex angle distributions used by FM are crucial when computing NMR parameters that are sensitive to short-range details of the pair distribution function, such as residual dipolar couplings, paramagnetic relaxation enhancement, or *J* coupling. However, upon closer examination, the pair-distance distribution generated by FM and a simpler model presented in this paper, globule-linker model (GLM; described below), become virtually identical for amino acids separated by >15 residues along the chain.

The highly localized differences between structures at small sequential distances have a minimal influence on the estimations of *R*_h_. It is important to recall that for amino acid residues separated by a distance *r*, the dipolar coupling decays as *r*^–3^, while the decay rate of hydrodynamic interactions (HI) is only *r*^–1^. Therefore, HI are long-range and less sensitive to near-neighbor distributions, with contributions to the diffusion coefficient of near neighbors and far neighbors being *O*(*N*) and *O*(*N*^2−γ^) = *O*(*N*^1.4^), respectively.

Guided by these considerations, we have implemented the simplest extension of Zimm’s chain, the globule-linker model (GLM), designed to comprehensively represent IDPs that contain globular domains connected by unstructured fragments. In particular, the GLM approach reflects the idea that the hydrodynamic radius corresponding to the experimentally measured long-time diffusion coefficient can be predicted under a minimal model that incorporates knowledge of domain boundaries in long protein chains and excluded volume interactions. In the model (Figure 1A–C), we represented the protein as an assembly of spheres of different sizes. Within the GLM approach, the conformational sampling is split into four stages: selection of domain boundaries, computation of steric radii of approximating spheres for globular domains, generation of locations of the domains and linkers, and addition of the hydration layer to the linkers.

**Figure 1:**
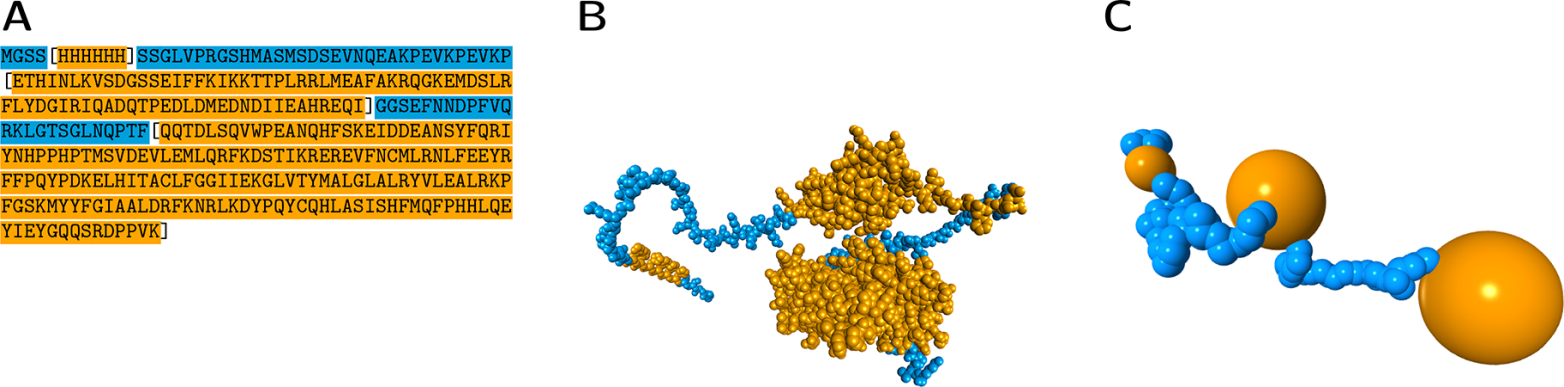
Construction of the coarse-grained globule-linker model (GLM) for an illustratory IDP, H_6_–SUMO–CNOT1(800–999), containing three ordered domains of different sizes (no. 28 in Table S1). (A) Sequence with highlighted ordered (orange) and disordered (blue) segments, and domain boundaries marked by square brackets. (B) Representative full atom conformation generated by AlphaFold2 (for visualization purposes only;^69,70^ beads with van der Waals radii; hydrogen atoms omitted for the sake of clarity). Ordered clusters (orange) form dense blobs connected with linkers (blue). (C) Visualization of a representative configuration generated using the GLM method in which beads are displayed with their hydrodynamic radii.

First, the protein sequence fragments to be treated as folded domains and mimicked by larger beads within GLM are selected using disorder probability *P* predicted by Disopred3.^71^ A fragment is assumed to be ordered if the *P* value is <50% for at least three subsequent amino acid residues, and the ordered fragments within a single folded domain can be linked by loops, whose length does not exceed 14 residues.^72^ Because Disopred3 has been trained on the experimental data sets to obtain position-specific scores calculated for each amino acid residue,^71^ the *P* value involves implicitly the sequence specificity, reflecting the intramolecular interactions responsible for domain folding. Together with taking into account the experimentally established limit for the loop length,^72^ this approach enables us to create a biochemically relevant semiempirical model of globular domain boundaries. Such a globule boundary-annotated amino acid sequence is passed to the next stage of the modeling pipeline.

Second, the steric sizes of the approximating beads are computed. The structured domains are represented by a single larger sphere each, with the size depending on their mass *m* computed with the equation *R*_h_ = (3*m*/4*πρ*_globular_)^1/3^ + *a*_hydration_, where ρ_globular_ = 0.52 Da/Å^3^,^73^ with a single layer hydration shell taken to be *a*_hydration_ = 3 Å thick. In the case of unstructured linkers between the domains, the beads representing amino acid residues of the linker are presumed to be indistinguishable. The composition of such linker sequences is known to be statistically biased toward the disorder-promoting residues (Pro, hydrophilic and charged residues) and deficient in hydrophobic and aromatic residues.^74,75^ The significance of the composition–conformation relationship was analyzed for IDPs in great detail in terms of polar, polyampholytic, and polyelectrolytic tracts with different charge patterning (reviewed by Das et al.^75^). Although it is clear that the dimensions of the charged IDP as a whole can be significantly influenced by electrostatic interactions depending on the solution conditions^7^ or charge patterning,^76^ it seems reasonable to assume that, in solutions providing both sufficient hydration and ionic strength, the interactions between the polar and charged residues within the unstructured linker become less pronounced due to effective screening, and the exact pairwise potentials between the linker residues can be neglected. Each unstructured segment of length *N* is thus modeled as a chain of *N* identical spheres, each with a diameter equal to the C_α_–C_α_ distance, and we obtain a list of steric radii of beads, which is passed on to the next modeling step.

Third, the centers of the beads are randomly sampled according to a generalization of a self-avoiding random walk. The distribution can be defined by first considering an auxiliary distribution of random walks of chains of spheres defined by demanding that distances between the centers of consecutive spheres along the chains are equal to the sum of their respective radii, and that each vector joining centers of adjacent spheres has a spherically uniform distribution. We then define the self-avoiding random walk of spheres (SARWS) to have the sphere centers distributed according to the random walk of spheres, conditional on the absence of self-intersections. Sampling from this distribution is achieved by a recursive algorithm described in the Supporting Information, which offers accelerated sampling as compared to a one-by-one randomization. The SARWS algorithm ensures that the excluded volume of the chain is accounted for.

The fourth and final stage of the conformer generation process takes in the locations of the centers of the spheres generated in the previous step and adjusts their size to better reflect the hydrodynamic thickness of the linkers. We transformed the sampled conformations into a hydrodynamic model by increasing bead sizes in the disordered fragments of generated conformations to an *R*_disordered_ of 4.2 Å, corresponding to the median value for all amino acids.^77^ In the resulting hydrodynamic model of linkers, the neighboring beads show substantial overlaps, requiring a careful treatment of the mobility matrices (see ref (78) for details). Note that the value of *R*_disordered_ has an only minor impact on the final results, because the hydrodynamic radius of long slender filaments depends logarithmically on their thickness.^79−82^

To compute *R*_h_ from the estimated ensembles, we have implemented two algorithms: the Kirkwood formula and the minimum dissipation approximation (MDA) method of Cichocki et al.^59^ Within the first approach,^83^ the hydrodynamic radius of a macromolecule is approximated by
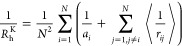
1where *N* is the total number of beads in the IDP model, *a*_*i*_ is the hydrodynamic radius of bead *i*, *r*_*ij*_ = |**r**_*j*_ – **r**_*i*_| is the distance between beads *i* and *j*, and the angle brackets denote the average over the equilibrium ensemble. One can show that this corresponds to the ensemble-averaged short-time diffusion coefficient of the geometric center of the macromolecule, **r**_c_ = *N*^–1^∑_*i*=1_^*N*^**r**_*i*_. Note that the geometric center fluctuates as the shape of the molecule evolves and does not correspond to any fixed position within it. A simplified form of the Kirkwood formula is often used^42,84,85^
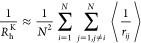
2where the single-bead terms 1/*a*_*i*_ are dropped, as their contribution becomes negligible in the large *N* limit. This is the form that we will use in the work presented here.

A better estimate of *R*_h_, corresponding to the long-time diffusion coefficient, requires a more in-depth description of the hydrodynamic interactions between the beads. To this end, one introduces mobility matrix **μ**,^54^ which links the velocities of the beads with the forces acting on them, according to
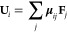
3where **U**_*i*_ is the velocity of bead *i*, whereas **F**_*j*_ is the force with which bead *j* acts on the fluid. On the basis of the mobility matrix, one defines a matrix **A** indexed by the bead labels (*i*, *j*), *A*_*ij*_ = 2*πη*Tr⟨**μ**_*ij*_⟩ and its inverse **B** = **A**^–1^. One can then construct the MDA^59^ for *R*_h_ as
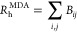
4Note that eq 4 is general and can be used for different models of hydrodynamic interactions, both simple models (e.g., Rotne–Prager far-field approximation^86^) and more sophisticated approaches, like the multipole expansion method.^87,88^ In this work, we use the generalized Rotne–Prager approximation to calculate the mobility matrix, as described in refs (89−91). This approximation is now also available as a Python package, pygrpy.^92^ For non-overlapping beads, the elements of matrix **A** have then a particularly simple form: *A*_*ij*_ = ⟨1/*r*_*ij*_⟩ for *i* ≠ *j*, and *A*_*ii*_ = 1/*a*_*i*_. The formulas for overlapping beads can be found in the Supporting Information.

The MDA corresponds to the calculation of the short-time diffusion coefficient of the diffusion center of a molecule,^58^ which is a point inside the molecule where *D*_s_ is minimal. The position of the diffusion center is **r**_d_ = ∑_*i*=1_^*N*^*x*_*i*_**r**_*i*_, with the weights given by *x*_*i*_ = ∑_*j*_*B*_*ij*_/∑_*k*,*j*_*B*_*kj*_. Because *D*_s_ is always larger than its long-time counterpart, *D*_l_, MDA provides the best estimation for the long-time diffusion coefficient of all of the methods that utilize *D*_s_ for this purpose. The MDA turns out to be more robust when dealing with large differences in the sizes of beads used to model constituent parts of the macromolecule, because in such cases the equal weights of the geometric center of the macromolecule used in the Kirkwood formula differ significantly from the optimal weights of the diffusion center.

We combined each method of generating conformers with each method of computing *R*_h_, which resulted in four different theoretical approaches, the predictions of which (Table S2) were then compared with experimental data. For this purpose, we have obtained 15 new IDP constructs covering a wide range of chain lenghts, folded domain contents, and charge states and determined their *R*_h_ using FCS (Figure 2 and Figures S2–S6; for further experimental details, see the Supporting Information).

**Figure 2:**
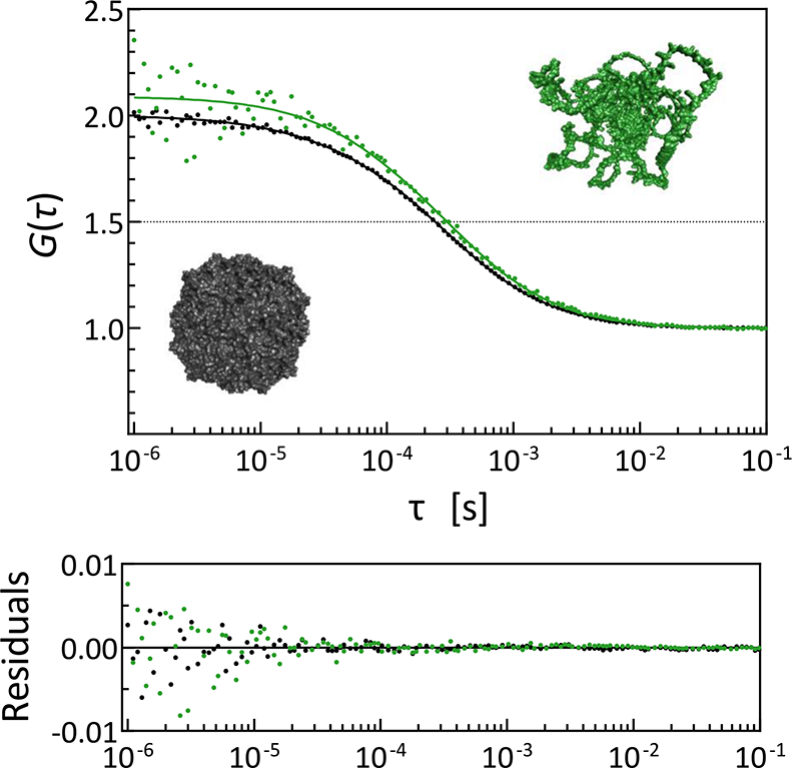
Examples of normalized FCS autocorrelation curves with raw fitting residuals for an intrinsically disordered H_6_–SUMO–GW182SD–mCherry (*N* = 809; *R*_h_ = 66 ± 6 Å) (green) in comparison with apoferritin (*N* = 4200; *R*_h_ = 58 ± 3 Å) (black). The crystal structure of apoferritin (Protein Data Bank entry 2w0o(93)) and the putative conformation of H_6_–SUMO–GW182SD–mCherry predicted by AlphaFold^69^ are shown for the purpose of illustration, preserving the relative sizes of the solvent accessible surfaces of atoms.

The experimental benchmark set (Table S1) was thus composed of both the new FCS measurements and *R*_h_ values selected from the literature on the basis of the following criteria. The proteins had sequences that could be unambiguously identified in the literature or in the UniProtKB database and were measured under well-defined, mild conditions (temperature of 20–26 °C, buffer of pH 7–8, and ionic strength corresponding to 75–300 mM NaCl), and their hydrodynamic radii were determined directly from appropriate experiments without conversions from other experimental quantities, such as *R*_g_.^29,94−111^ This is, to our best knowledge, the largest benchmark set encompassing experimental *R*_h_ values for 38 IDPs and six globular model proteins, measured under comparable conditions (Figure 3).

**Figure 3:**
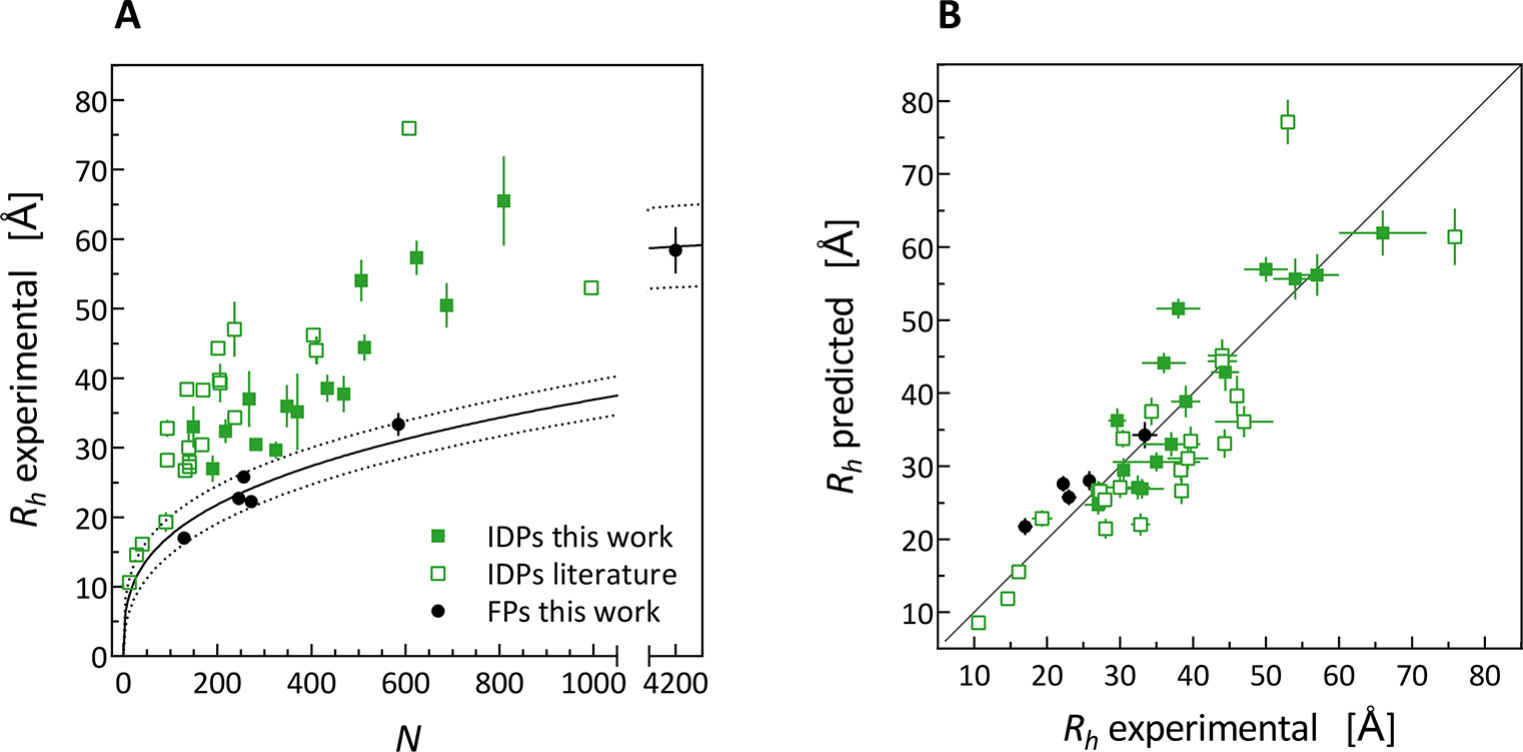
(A) Experimental *R*_h_ values plotted vs the number of amino acid residues in the protein chain, *N*, and power law curve fitted to *R*_h_ values of folded proteins (FPs) together with the 95% confidence band. (B) Direct comparison of the predicted vs measured *R*_h_ values for all of the proteins modeled using the MDA+GLM approach.

The results of tests performed for our four theoretical approaches against the benchmark set are listed in Table 1, and Figure 4 shows a visual comparison of the deviations between theory and experiment. Additionally, we provide various power law fits^112−114^ for comparison of the prediction accuracy (Table S3).

**Table 1:** Comparison of Error Statistics of Various Models^a^

model	*n*_fp_	RMSD (Å)	RMSRD (%)	*R*^2^	*R*^2^_adj_	*Q*_3_^AE^ (Å)	*Q*_3_^RE^ (%)
MDA+GLM	0	7.09	18.15	0.71	0.71	6.80	22.51
MDA+GLM(ND)	0	9.48	28.02	0.48	0.48	11.88	29.31
KR+GLM	0	12.82	34.69	0.05	0.05	17.59	42.95
KR+GLM(ND)	0	9.25	27.31	0.50	0.50	11.11	29.44
random coil	1	9.60	27.71	0.47	0.45	10.28	33.69
power law	2	8.46	24.80	0.59	0.56	9.63	26.08
power law (ref (112))	2	12.01	36.94	0.16	0.12	14.37	39.51
PPII-based (ref (30))	3	17.25	49.09	–0.72	–0.86	20.62	59.54
PPII and |*Q*|-based (ref (31))	5	18.97	47.71	–1.08	–1.36	19.60	54.77
sequence-based (ref (112))	7	22.90	50.78	–2.05	–2.66	19.59	58.32

*a* *n*_fp_ is the number of fitting parameters. ND indicates no domain information. *Q*_3_ is the third quartile.

**Figure 4:**
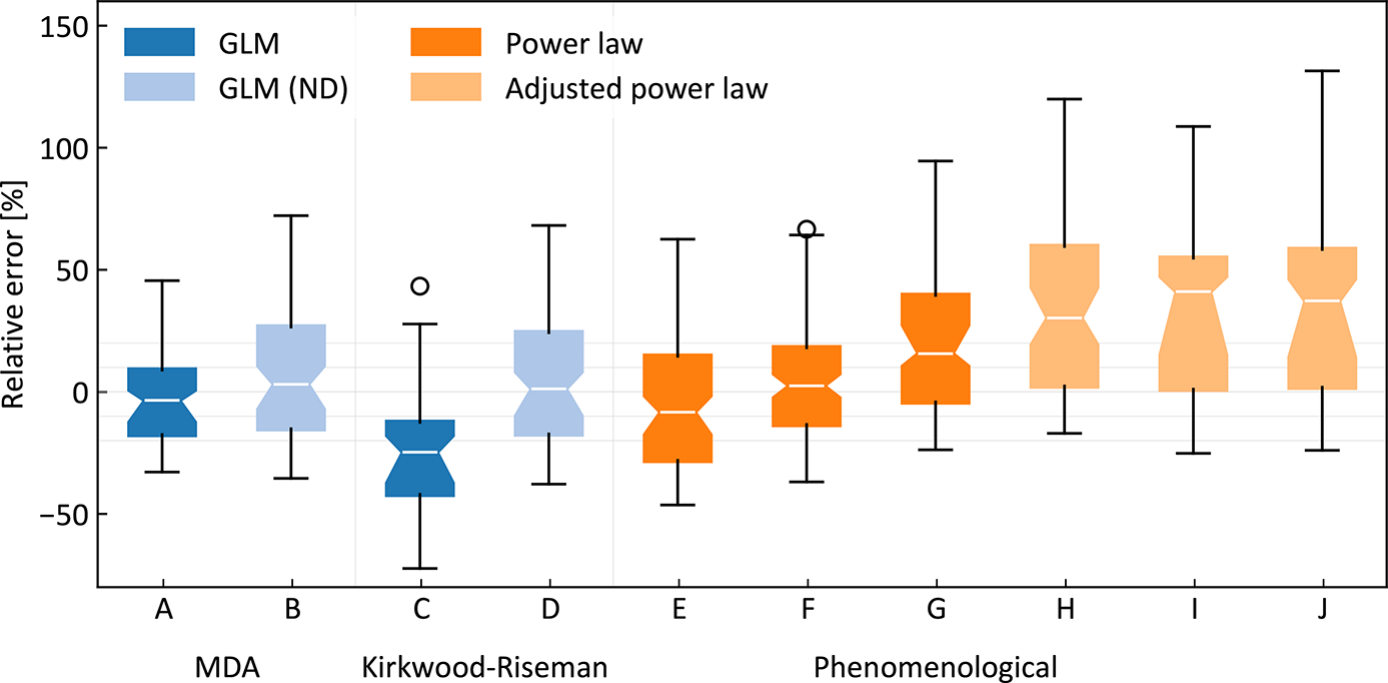
Comparison of different methods of estimation of *R*_h_. Boxes show interquartile ranges with median confidence bands marked by notches. MDA with GLM ensemble generation (A) performs best on the IDP benchmark set with standard errors of 18.15% and 7.09 Å (compared to 24.80% and 8.46 Å for a simple power law). Methods based on the Kirkwood–Riseman *R*_h_ estimation (C and D) typically underestimate hydrodynamic size of the molecule. Power law fits with one free parameter (E) and two free parameters (F) evaluated using leave-one-out cross-validation are compared with the formerly reported power law^112^ (G) and models based on polyproline II structure propensities without^30^ (H) and with^31^ (I) regard to the charge, and a sequence-based model^112^ (J) that takes into account the total charge of the molecule. Theoretical methods with no knowledge of the presence of domains in the IDP (ND; B and D) significantly overestimate the hydrodynamic size of the molecule. Domain data can be incorporated into our ensemble generation engine leading to more accurate estimates of *R*_h_ (A). Note that experimental uncertainty also contributes to the errors presented here and in Table 1.

We compare the accuracy of the previous and new model under six metrics (Table 1): the square root of the mean square deviation (RMSD), the square root of the mean square relative deviation (RMSRD), Pearson’s coefficient (*R*^2^), Pearson’s coefficient adjusted for fitting parameters (*R*^2^_adj_), the third quartile of the absolute error (*Q*_3_^AE^), and the third quartile of the relative error (*Q*_75_^RE^). Whenever a fitting procedure is required, we use leave-one-out cross-validation to compute error metrics. We also have chosen to test the relative deviations to reduce the undue weight given to the new, very long sequences in our data set. Similarly, outlier-robust metrics of the third quartile were included to reduce the impact of a single-sequence misprediction on the final comparisons. In all evaluation metrics, the MDA+GLM approach performs the best. Surprisingly, it is the only model that performs better than the power law baseline in any of the evaluation metrics.

Interestingly, it is apparent from the comparison of the results obtained using MDA+GLM with those from MDA+GLM(ND) in Table 1 and Figure 4 (A and B) that the proper identification of the globular domain boundaries proves to be the main condition for successfully estimating the *R*_h_ value of an IDP, with better accuracy than all other tested approaches. This means that the pairwise interactions between the linker amino acid residues influence *R*_h_ to a lesser extent, while the sizes of the globular domains and their relative spatial distribution are very important.

It should be mentioned, however, that a significant contribution to the discrepancies between the experimental and predicted *R*_h_ values (Figure S8) comes from the intrinsic properties of the individual experimental methods, which suffer from typical errors or limitations and are usually not taken into account when reporting the final experimental results. PGF-NMR measurements are the most unambiguous and accurate, but their effective application is limited to smaller proteins (up to 200–300 amino acid residues long) at high concentrations. It is worth noting that the agreement of the values of *R*_h_ predicted by MDA+GLM with the PGF-NMR results is excellent (Figure S8C). FCS is the only method that addresses the self-diffusion of molecules at the low-concentration limit. Raw FCS measurements can be refined to exclude possible oligomerization or aggregation during the experiment on the basis of the count rates, but it is impossible to avoid proteolytic instability of proteins and, consequently, the appearance of impurities with a lower molar mass, which may potentially result in apparently lower values of *R*_h_ (Figure S8B). On the contrary, SEC is the easiest approach for removing lower-mass impurities, but it involves diffusion of molecules at higher concentrations through a medium with pores of a specific shape under the influence of pressure. An additional common disadvantage is calibration based on *R*_h_ of standard proteins determined under various conditions and the lack of appropriate propagation of the calibration experimental uncertainty. Consequently, SEC measurements can be highly scattered (Figure S8D). The largest outlier in our analysis concerns *R*_h_ determined using SEC for fesselin without providing experimental uncertainty (Id. 43, Tables S1 and S2 and Figures S7 and S8D). The DLS method is the most prone to overestimating experimental values (Figure S8E), because the presence of even a small number of aggregates with a larger molar mass generates a huge contribution to the intensity of scattered light. Finally, AUC yields sedimentation coefficients, and their interpretation in terms of exact values of *R*_h_ requires some assumptions that are not obvious for IDPs, such as, e.g., partial specific protein volume.^115^ The second largest outlier in our set is the OMM-64 protein (Id. 39, Tables S1 and S2 and Figures S7 and S8F) with the *R*_h_ value determined using AUC, which is very close to the power law curve for completely denatured proteins.^116^

In conclusion, we have presented a simple, first-principles model for the prediction of *R*_h_ without any fitting parameters and achieved favorable comparison with a large benchmark set. The sizes and positions of the globular domains proved to be the dominating factors that influence the hydrodynamic properties of the IDP chain as a whole. Moreover, due to the relative simplicity of the model, all of the calculations for a given protein can be performed in ∼1 min on a typical laptop, which is contrasted with MD simulation-based conformer generation methods that require supercomputers and take many days. Moreover, the MDA+GLM approach demonstrates satisfactory convergence even with ensemble sizes as small as 40 conformers (Figure S1).

Our benchmark set, in which the previously known IDPs were complemented by a set of newly obtained proteins, constitutes a significant step forward in predicting the hydrodynamic properties of IDPs. It includes a higher degree of conformational variety, with a stronger emphasis on multidomain proteins, longer chains, and a much wider range of charge states compared to the reference sets used previously.^30,112^ This diversity allows for more reliable testing of theoretical models. In particular, the presence of large polyanionic proteins in our set revealed that the *R*_h_ values obtained using phenomenological models corrected to account for the absolute net charge seem to be overestimated [Figure 4 (I and J)].

The sequence specificity effects are neglected in our model for the linker fragments, which is one of the possible sources of uncertainty. However, in our opinion, it is an acceptable level of error for such a quick numerical method. Further developments of the MDA+GLM approach are needed to take into account the dependence of *R*_h_ on the environmental conditions^6−8^ and the formation of complexes. More subtle effects related to the conformational properties of the linkers can be also included using sequence-based conformational ensembles.^45,47,117^ Nevertheless, our results demonstrate that the relatively simple globule-linker model for conformational ensemble construction, in combination with the minimum dissipation approximation, can serve as the starting point for developing further phenomenological corrections. These improvements could incorporate factors such as amino acid sequence composition, residue charge, and counterion binding. When using the MDA+GLM approach, all excluded volume effects are already correctly accounted for, with any further deviations hinting at the interesting physical and chemical properties of the molecules.

## Supporting Information Available

Experimental *R*_h_ values, Figures S1–S8, and additional methodological details (PDF)
Theoretical *R*_h_ values from GLM, phenomenological *R*_h_ values, and protein sequences with marked domains (Tables S2–S4) (XLSX)
Transparent Peer Review report available (PDF)

## Notes

The programs referenced in this article can be found on GitHub. For the sake of convenience, an API and a command-line Python utility, glm_mda_diffusion, have been provided.^118^ This package builds upon previous ones, namely, pychastic and sarw_spheres, both of which are also accessible on GitHub.

## References

[ref1] Oldfield C. J.; Dunker A. K. Intrinsically disordered proteins and intrinsically disordered protein regions. Annu. Rev. Biochem. 2014, 83, 553–584. 10.1146/annurev-biochem-072711-164947. 24606139

[ref2] Wright P. E.; Dyson H. J. Intrinsically disordered proteins in cellular signalling and regulation. Nat. Rev. Mol. Cell Biol. 2015, 16, 18–29. 10.1038/nrm3920. 25531225 PMC4405151

[ref3] Ward J. J.; Sodhi J. S.; McGuffin L. J.; Buxton B. F.; Jones D. T. Prediction and functional analysis of native disorder in proteins from the three kingdoms of life. J. Mol. Biol. 2004, 337, 635–645. 10.1016/j.jmb.2004.02.002. 15019783

[ref4] Shea J.-E.; Best R. B.; Mittal J. Physics-based computational and theoretical approaches to intrinsically disordered proteins. Curr. Opin. Struct. Biol. 2021, 67, 219–225. 10.1016/j.sbi.2020.12.012. 33545530 PMC8150118

[ref5] Uversky V. N. Intrinsically disordered proteins and their environment: effects of strong denaturants, temperature, pH, counter ions, membranes, binding partners, osmolytes, and macromolecular crowding. Protein Journal 2009, 28, 305–325. 10.1007/s10930-009-9201-4. 19768526

[ref6] Langridge T. D.; Tarver M. J.; Whitten S. T. Temperature effects on the hydrodynamic radius of the intrinsically disordered N-terminal region of the p53 protein. Proteins: Struct., Funct., Bioinf. 2014, 82, 668–678. 10.1002/prot.24449. 24150971

[ref7] Müller-Späth S.; Soranno A.; Hirschfeld V.; Hofmann H.; Rüegger S.; Reymond L.; Nettels D.; Schuler B. Charge interactions can dominate the dimensions of intrinsically disordered proteins. Proc. Natl. Acad. Sci. U. S. A. 2010, 107, 14609–14614. 10.1073/pnas.1001743107. 20639465 PMC2930438

[ref8] Wohl S.; Jakubowski M.; Zheng W. Salt-dependent conformational changes of intrinsically disordered proteins. J. Phys. Chem. Lett. 2021, 12, 6684–6691. 10.1021/acs.jpclett.1c01607. 34259536

[ref9] Moses D.; Yu F.; Ginell G. M.; Shamoon N. M.; Koenig P. S.; Holehouse A. S.; Sukenik S. Revealing the hidden sensitivity of intrinsically disordered proteins to their chemical environment. J. Phys. Chem. Lett. 2020, 11, 10131–10136. 10.1021/acs.jpclett.0c02822. 33191750 PMC8092420

[ref10] Wang Y.; Benton L. A.; Singh V.; Pielak G. J. Disordered protein diffusion under crowded conditions. J. Phys. Chem. Lett. 2012, 3, 2703–2706. 10.1021/jz3010915. 23185649 PMC3505085

[ref11] Bah A.; Vernon R. M.; Siddiqui Z.; Krzeminski M.; Muhandiram R.; Zhao C.; Sonenberg N.; Kay L. E.; Forman-Kay J. D. Folding of an intrinsically disordered protein by phosphorylation as a regulatory switch. Nature 2015, 519, 106–109. 10.1038/nature13999. 25533957

[ref12] Vancraenenbroeck R.; Harel Y. S.; Zheng W.; Hofmann H. Polymer effects modulate binding affinities in disordered proteins. Proc. Natl. Acad. Sci., U. S. A. 2019, 116, 19506–19512. 10.1073/pnas.1904997116. 31488718 PMC6765308

[ref13] Borgia A.; Borgia M. B.; Bugge K.; Kissling V. M.; Heidarsson P. O.; Fernandes C. B.; Sottini A.; Soranno A.; Buholzer K. J.; Nettels D.; et al. Extreme disorder in an ultrahigh-affinity protein complex. Nature 2018, 555, 61–66. 10.1038/nature25762. 29466338 PMC6264893

[ref14] Seiffert P.; Bugge K.; Nygaard M.; Haxholm G. W.; Martinsen J. H.; Pedersen M. N.; Arleth L.; Boomsma W.; Kragelund B. B. Orchestration of signaling by structural disorder in class 1 cytokine receptors. Cell Commun. Signaling 2020, 18, 132 10.1186/s12964-020-00626-6. PMC7444064 32831102

[ref15] Evans J. S. The biomineralization proteome: protein complexity for a complex bioceramic assembly process. Proteomics 2019, 19, 1900036 10.1002/pmic.201900036. 31219243

[ref16] Krois A. S.; Dyson H. J.; Wright P. E. Long-range regulation of p53 DNA binding by its intrinsically disordered N-terminal transactivation domain. Proc. Natl. Acad. Sci. U. S. A. 2018, 115, E11302–E11310. 10.1073/pnas.1814051115. 30420502 PMC6275486

[ref17] Fletcher C. M.; McGuire A. M.; Gingras A.-C.; Li H.; Matsuo H.; Sonenberg N.; Wagner G. 4E binding proteins inhibit the translation factor eIF4E without folded structure. Biochemistry 1998, 37, 9–15. 10.1021/bi972494r. 9453748

[ref18] Fletcher C. M.; Wagner G. The interaction of eIF4E with 4E-BP1 is an induced fit to a completely disordered protein. Protein Sci. 1998, 7, 1639–1642. 10.1002/pro.5560070720. 9684899 PMC2144065

[ref19] Gingras A.-C.; Raught B.; Gygi S. P.; Niedzwiecka A.; Miron M.; Burley S. K.; Polakiewicz R. D.; Wyslouch-Cieszynska A.; Aebersold R.; Sonenberg N. Hierarchical phosphorylation of the translation inhibitor 4E-BP1. Genes Dev. 2001, 15, 2852–2864. 10.1101/gad.912401. 11691836 PMC312813

[ref20] Sheu-Gruttadauria J.; MacRae I. J. Phase transitions in the assembly and function of human miRISC. Cell 2018, 173, 946–957. 10.1016/j.cell.2018.02.051. 29576456 PMC5935535

[ref21] Cieplak-Rotowska M. K.; Tarnowski K.; Rubin M.; Fabian M. R.; Sonenberg N.; Dadlez M.; Niedzwiecka A. Structural dynamics of the GW182 silencing domain including its RNA recognition motif (RRM) revealed by hydrogen-deuterium exchange mass spectrometry. Journal of The American Society for Mass Spectrometry 2018, 29, 158–173. 10.1007/s13361-017-1830-9. 29080206 PMC5785596

[ref22] Raisch T.; Valkov E. Regulation of the multisubunit CCR4-NOT deadenylase in the initiation of mRNA degradation. Curr. Opin. Struct. Biol. 2022, 77, 102460 10.1016/j.sbi.2022.102460. 36116370 PMC9771892

[ref23] Louros N.; Schymkowitz J.; Rousseau F. Mechanisms and pathology of protein misfolding and aggregation. Nat. Rev. Mol. Cell Biol. 2023, 24, 912–933. 10.1038/s41580-023-00647-2. 37684425

[ref24] Chakraborty P.; Zweckstetter M. Role of aberrant phase separation in pathological protein aggregation. Curr. Opin. Struct. Biol. 2023, 82, 102678 10.1016/j.sbi.2023.102678. 37604044

[ref25] Banani S. F.; Rice A. M.; Peeples W. B.; Lin Y.; Jain S.; Parker R.; Rosen M. K. Compositional control of phase-separated cellular bodies. Cell 2016, 166, 651–663. 10.1016/j.cell.2016.06.010. 27374333 PMC4967043

[ref26] Forman-Kay J. D.; Ditlev J. A.; Nosella M. L.; Lee H. O. What are the distinguishing features and size requirements of biomolecular condensates and their implications for RNA-containing condensates?. RNA 2022, 28, 36–47. 10.1261/rna.079026.121. 34772786 PMC8675286

[ref27] Białobrzewski M. K.; Klepka B. P.; Michaś A.; Cieplak-Rotowska M. K.; Staszałek Z.; Niedźwiecka A. Diversity of hydrodynamic radii of intrinsically disordered proteins. Eur. Biophys. J. 2023, 52, 607–618. 10.1007/s00249-023-01683-8. 37831084 PMC10618399

[ref28] Nygaard M.; Kragelund B. B.; Papaleo E.; Lindorff-Larsen K. An efficient method for estimating the hydrodynamic radius of disordered protein conformations. Biophys. J. 2017, 113, 550–557. 10.1016/j.bpj.2017.06.042. 28793210 PMC5550300

[ref29] Pesce F.; Newcombe E. A.; Seiffert P.; Tranchant E. E.; Olsen J. G.; Grace C. R.; Kragelund B. B.; Lindorff-Larsen K. Assessment of models for calculating the hydrodynamic radius of intrinsically disordered proteins. Biophys. J. 2023, 122, 310–321. 10.1016/j.bpj.2022.12.013. 36518077 PMC9892621

[ref30] Tomasso M. E.; Tarver M. J.; Devarajan D.; Whitten S. T. Hydrodynamic radii of intrinsically disordered proteins determined from experimental polyproline II propensities. PLoS Computational Biology 2016, 12, e1004686 10.1371/journal.pcbi.1004686. 26727467 PMC4699819

[ref31] English L. R.; Tilton E. C.; Ricard B. J.; Whitten S. T. Intrinsic α helix propensities compact hydrodynamic radii in intrinsically disordered proteins. Proteins: Struct., Funct., Bioinf. 2017, 85, 296–311. 10.1002/prot.25222. PMC5258847 27936491

[ref32] Song J.; Li J.; Chan H. S. Small-Angle X-ray Scattering Signatures of Conformational Heterogeneity and Homogeneity of Disordered Protein Ensembles. J. Phys. Chem. B 2021, 125, 6451–6478. 10.1021/acs.jpcb.1c02453. 34115515

[ref33] Song J.; Gomes G.-N.; Gradinaru C. C.; Chan H. S. An Adequate Account of Excluded Volume Is Necessary To Infer Compactness and Asphericity of Disordered Proteins by Förster Resonance Energy Transfer. J. Phys. Chem. B 2015, 119, 15191–15202. 10.1021/acs.jpcb.5b09133. 26566073

[ref34] Liu B.; Chia D.; Csizmok V.; Farber P.; Forman-Kay J. D.; Gradinaru C. C. The Effect of Intrachain Electrostatic Repulsion on Conformational Disorder and Dynamics of the Sic1 Protein. J. Phys. Chem. B 2014, 118, 4088–4097. 10.1021/jp500776v. 24673507

[ref35] Mao A. H.; Crick S. L.; Vitalis A.; Chicoine C. L.; Pappu R. V. Net charge per residue modulates conformational ensembles of intrinsically disordered proteins. Proc. Natl. Acad. Sci. U. S. A. 2010, 107, 8183–8188. 10.1073/pnas.0911107107. 20404210 PMC2889596

[ref36] Różycki B.; Kim Y. C.; Hummer G. SAXS ensemble refinement of ESCRT-III CHMP3 conformational transitions. Structure 2011, 19, 109–116. 10.1016/j.str.2010.10.006. 21220121 PMC3032427

[ref37] Ozenne V.; Bauer F.; Salmon L.; Huang J.-r.; Jensen M. R.; Segard S.; Bernadó P.; Charavay C.; Blackledge M. Flexible-meccano: a tool for the generation of explicit ensemble descriptions of intrinsically disordered proteins and their associated experimental observables. Bioinformatics 2012, 28, 1463–1470. 10.1093/bioinformatics/bts172. 22613562

[ref38] Mittal J.; Yoo T. H.; Georgiou G.; Truskett T. M. Structural ensemble of an intrinsically disordered polypeptide. J. Phys. Chem. B 2013, 117, 118–124. 10.1021/jp308984e. 23205890

[ref39] Mittal A.; Holehouse A. S.; Cohan M. C.; Pappu R. V. Sequence-to-conformation relationships of disordered regions tethered to folded domains of proteins. J. Mol. Biol. 2018, 430, 2403–2421. 10.1016/j.jmb.2018.05.012. 29763584

[ref40] Das P.; Matysiak S.; Mittal J. Looking at the disordered proteins through the computational microscope. ACS Central Science 2018, 4, 534–542. 10.1021/acscentsci.7b00626. 29805999 PMC5968442

[ref41] Estaña A.; Sibille N.; Delaforge E.; Vaisset M.; Cortés J.; Bernadó P. Realistic ensemble models of intrinsically disordered proteins using a structure-encoding coil database. Structure 2019, 27, 381–391. 10.1016/j.str.2018.10.016. 30554840

[ref42] Baul U.; Chakraborty D.; Mugnai M. L.; Straub J. E.; Thirumalai D. Sequence effects on size, shape, and structural heterogeneity in intrinsically disordered proteins. J. Phys. Chem. B 2019, 123, 3462–3474. 10.1021/acs.jpcb.9b02575. 30913885 PMC6920032

[ref43] Garcia de la Torre J.; Hernández Cifre J. G. Hydrodynamic properties of biomacromolecules and macromolecular complexes: concepts and methods. A tutorial mini-review. J. Mol. Biol. 2020, 432, 2930–2948. 10.1016/j.jmb.2019.12.027. 31877325

[ref44] Gomes G.-N. W.; Krzeminski M.; Namini A.; Martin E. W.; Mittag T.; Head-Gordon T.; Forman-Kay J. D.; Gradinaru C. C. Conformational ensembles of an intrinsically disordered protein consistent with NMR, SAXS, and single-molecule FRET. J. Am. Chem. Soc. 2020, 142, 15697–15710. 10.1021/jacs.0c02088. 32840111 PMC9987321

[ref45] Tesei G.; Schulze T. K.; Crehuet R.; Lindorff-Larsen K. Accurate model of liquid-liquid phase behavior of intrinsically disordered proteins from optimization of single-chain properties. Proc. Natl. Acad. Sci. U. S. A. 2021, 118, e2111696118 10.1073/pnas.2111696118. 34716273 PMC8612223

[ref46] Tesei G.; Trolle A. I.; Jonsson N.; Betz J.; Knudsen F. E.; Pesce F.; Johansson K. E.; Lindorff-Larsen K. Conformational ensembles of the human intrinsically disordered proteome. Nature 2024, 626, 897–904. 10.1038/s41586-023-07004-5. 38297118

[ref47] Lotthammer J. M.; Ginell G. M.; Griffith D.; Emenecker R. J.; Holehouse A. S. Direct prediction of intrinsically disordered protein conformational properties from sequence. Nat. Methods 2024, 21, 465–476. 10.1038/s41592-023-02159-5. 38297184 PMC10927563

[ref48] Zimm B. H. Chain molecule hydrodynamics by the Monte-Carlo method and the validity of the Kirkwood-Riseman approximation. Macromolecules 1980, 13, 592–602. 10.1021/ma60075a022.

[ref49] Zimm B. H. Sedimentation of asymmetric elastic dumbbells and the rigid-body approximation in the hydrodynamics of chains. Macromolecules 1982, 15, 520–525. 10.1021/ma00230a059.

[ref50] Rodriguez Schmidt R.; Hernández Cifre J. G.; Garcia de la Torre J. Translational diffusion coefficients of macromolecules. Eur. Phys. J. E 2012, 35, 130 10.1140/epje/i2012-12130-x. 23239268

[ref51] de la Torre J. G. Analytical Ultracentrifugation. Instrumentation, Software, and Applications; Springer 2016, 195–217. 10.1007/978-4-431-55985-6_11. 27466593

[ref52] Fixman M. Inclusion of hydrodynamic interaction in polymer dynamical simulations. Macromolecules 1981, 14, 1710–1717. 10.1021/ma50007a019.

[ref53] Fixman M. Variational bounds for polymer transport coefficients. J. Chem. Phys. 1983, 78, 1588–1593. 10.1063/1.444849.

[ref54] Happel J.; Brenner H. Low Reynolds Number Hydrodynamics; Noordhoff: Leiden, The Netherlands, 1973.

[ref55] Cichocki B.; Ekiel-Jeżewska M. L.; Wajnryb E. Communication: Translational Brownian motion for particles of arbitrary shape. J. Chem. Phys. 2012, 136, 071102 10.1063/1.3689842. 22360229

[ref56] Wegener W. A. Bead models of segmentally flexible macromolecules. J. Chem. Phys. 1982, 76, 6425–6430. 10.1063/1.442999.

[ref57] Harvey S. C.; Mellado P.; García de la Torre J. Hydrodynamic resistance and diffusion coefficients of segmentally flexible macromolecules with two subunits. J. Chem. Phys. 1983, 78, 2081–2090. 10.1063/1.444917.

[ref58] Wegener W. A. Center of diffusion of flexible macromolecules. Macromolecules 1985, 18, 2522–2530. 10.1021/ma00154a029.

[ref59] Cichocki B.; Rubin M.; Niedzwiecka A.; Szymczak P. Diffusion coefficients of elastic macromolecules. J. Fluid Mech. 2019, 878, R3 10.1017/jfm.2019.652.

[ref60] Karplus M.; Petsko G. A. Molecular dynamics simulations in biology. Nature 1990, 347, 631–639. 10.1038/347631a0. 2215695

[ref61] Doi M.; Edwards S. F. The Theory of Polymer Dynamics; Oxford University Press, 1988; Vol. 73.

[ref62] Ravi Prakash J. The kinetic theory of dilute solutions of flexible polymers: Hydrodynamic interaction. Rheol. Ser. 1999, 8, 467–517. 10.1016/S0169-3107(99)80039-2.

[ref63] Szymczak P.; Cieplak M. Hydrodynamic effects in proteins. J. Phys.: Condens. Matter 2011, 23, 033102 10.1088/0953-8984/23/3/033102. 21406855

[ref64] Skolnick J. Perspective: On the importance of hydrodynamic interactions in the subcellular dynamics of macromolecules. J. Chem. Phys. 2016, 145, 100901 10.1063/1.4962258. 27634243 PMC5018002

[ref65] Frenkel D.; Smit B. Understanding molecular simulation: from algorithms to applications; Elsevier, 2001; Vol. 1.

[ref66] Rouse P. E. A Theory of the Linear Viscoelastic Properties of Dilute Solutions of Coiling Polymers. J. Chem. Phys. 1953, 21, 1272–1280. 10.1063/1.1699180.

[ref67] Zimm B. H. Journal of Chemical Physics. Dynamics of Polymer Molecules in Dilute Solution: Viscoelasticity, Flow Birefringence and Dielectric Loss 1956, 24, 269–278. 10.1063/1.1742462.

[ref68] Kirkwood J. G.; Riseman J. The intrinsic viscosities and diffusion constants of flexible macromolecules in solution. J. Chem. Phys. 1948, 16, 565–573. 10.1063/1.1746947.

[ref69] Jumper J.; Evans R.; Pritzel A.; Green T.; Figurnov M.; Ronneberger O.; Tunyasuvunakool K.; Bates R.; Žídek A.; Potapenko A.; et al. Highly accurate protein structure prediction with AlphaFold. Nature 2021, 596, 583–589. 10.1038/s41586-021-03819-2. 34265844 PMC8371605

[ref70] Ruff K. M.; Pappu R. V. AlphaFold and implications for intrinsically disordered proteins. J. Mol. Biol. 2021, 433, 167208 10.1016/j.jmb.2021.167208. 34418423

[ref71] Jones D. T.; Cozzetto D. DISOPRED3: precise disordered region predictions with annotated protein-binding activity. Bioinformatics 2015, 31, 857–863. 10.1093/bioinformatics/btu744. 25391399 PMC4380029

[ref72] Choi Y.; Agarwal S.; Deane C. M. How long is a piece of loop?. PeerJ. 2013, 1, e1 10.7717/peerj.1. 23638343 PMC3628373

[ref73] Murphy L. R.; Matubayasi N.; Payne V. A.; Levy R. M. Protein hydration and unfolding-insights from experimental partial specific volumes and unfolded protein models. Folding and Design 1998, 3, 105–118. 10.1016/S1359-0278(98)00016-9. 9565755

[ref74] Romero P.; Obradovic Z.; Li X.; Garner E. C.; Brown C. J.; Dunker A. K. Sequence complexity of disordered protein. Proteins: Struct., Funct., Bioinf. 2001, 42, 38–48. 10.1002/1097-0134(20010101)42:1<38::AID-PROT50>3.0.CO;2-3. 11093259

[ref75] Das R. K.; Ruff K. M.; Pappu R. V. Relating sequence encoded information to form and function of intrinsically disordered proteins. Curr. Opin. Struct. Biol. 2015, 32, 102–112. 10.1016/j.sbi.2015.03.008. 25863585 PMC4512920

[ref76] Lin Y.-H.; Chan H. S. Phase Separation and Single-Chain Compactness of Charged Disordered Proteins Are Strongly Correlated. Biophys. J. 2017, 112, 2043–2046. 10.1016/j.bpj.2017.04.021. 28483149 PMC5448239

[ref77] Longsworth L. Diffusion measurements, at 25°C, of aqueous solutions of amino acids, peptides and sugars. J. Am. Chem. Soc. 1953, 75, 5705–5709. 10.1021/ja01118a065.

[ref78] Zuk P.; Wajnryb E.; Mizerski K.; Szymczak P. Rotne-Prager-Yamakawa approximation for different-sized particles in application to macromolecular bead models. J. Fluid Mech. 2014, 741, R5 10.1017/jfm.2013.668.

[ref79] Cox R. The motion of long slender bodies in a viscous fluid Part 1. General theory. J. Fluid Mech. 1970, 44, 791–810. 10.1017/S002211207000215X.

[ref80] Johnson R. E.; Wu T. Y. Hydromechanics of low-Reynolds-number flow. Part 5. Motion of a slender torus. J. Fluid Mech. 1979, 95, 263–277. 10.1017/S0022112079001464.

[ref81] Majumdar S. R.; O’Neill M. E. On axisymmetric stokes flow past a torus. Zeitschrift für Angewandte Mathematik und Physik ZAMP 1977, 28, 541–550. 10.1007/BF01601334.

[ref82] Waszkiewicz R.; Szymczak P.; Lisicki M. Stability of sedimenting flexible loops. J. Fluid Mech. 2021, 919, A14 10.1017/jfm.2021.350.

[ref83] Kirkwood J. G. The general theory of irreversible processes in solutions of macromolecules. J. Polym. Sci. 1954, 12, 1–14. 10.1002/pol.1954.120120102.

[ref84] Liu B.; Dünweg B. Translational diffusion of polymer chains with excluded volume and hydrodynamic interactions by Brownian dynamics simulation. J. Chem. Phys. 2003, 118, 8061–8072. 10.1063/1.1564047.

[ref85] Vovk A.; Zilman A. Effects of Sequence Composition, Patterning and Hydrodynamics on the Conformation and Dynamics of Intrinsically Disordered Proteins. International Journal of Molecular Sciences 2023, 24, 1444 10.3390/ijms24021444. 36674958 PMC9867189

[ref86] Kim S.; Karrila S. J. Microhydrodynamics: Principles and Selected Applications; Butterworth-Heinemann: London, 1991.

[ref87] Mazur P.; van Saarloos W. Many-sphere hydrodynamic interactions and mobilities in a suspension. Physica A 1982, 115, 21–57. 10.1016/0378-4371(82)90127-3.

[ref88] Cichocki B.; Felderhof B. U.; Hinsen K.; Wajnryb E.; Bławzdziewicz J. Friction and mobility of many spheres in Stokes flow. J. Chem. Phys. 1994, 100, 3780–3790. 10.1063/1.466366.

[ref89] Rotne J.; Prager S. Variational treatment of hydrodynamic interaction in polymers. J. Chem. Phys. 1969, 50, 4831–4837. 10.1063/1.1670977.

[ref90] Yamakawa H. Transport properties of polymer chains in dilute solution: Hydrodynamic interaction. Jorunal of Chemical Physics 1970, 53, 436–443. 10.1063/1.1673799.

[ref91] Zuk P.; Cichocki B.; Szymczak P. GRPY: An Accurate Bead Method for Calculation of Hydrodynamic Properties of Rigid Biomacromolecules. Biophys. J. 2018, 115, 782–800. 10.1016/j.bpj.2018.07.015. 30144937 PMC6127458

[ref92] Waszkiewicz R.; Bartczak M.; Kolasa K.; Lisicki M. Pychastic: Precise Brownian dynamics using Taylor-Ito̅ integrators in Python. SciPost Physics Codebases 2023, 11, n/a 10.21468/SciPostPhysCodeb.11.

[ref93] de Val N.; Declercq J.-P.; Lim C. K.; Crichton R. R. Structural analysis of haemin demetallation by L-chain apoferritins. Journal of Inorganic Biochemistry 2012, 112, 77–84. 10.1016/j.jinorgbio.2012.02.031. 22561545

[ref94] McCubbin W. D.; Kay C. M.; Lane B. G. Hydrodynamic and optical properties of the wheat germ Em protein. Canadian Journal of Biochemistry and Cell Biology 1985, 63, 803–811. 10.1139/o85-102.

[ref95] Guez V.; Nair S.; Chaffotte A.; Bedouelle H. The anticodon-binding domain of tyrosyl-tRNA synthetase: state of folding and origin of the crystallographic disorder. Biochemistry 2000, 39, 1739–1747. 10.1021/bi992382v. 10677223

[ref96] Bouvier M.; Stafford W. F. Probing the three-dimensional structure of human calreticulin. Biochemistry 2000, 39, 14950–14959. 10.1021/bi0019545. 11101311

[ref97] Danielsson J.; Jarvet J.; Damberg P.; Gräslund A. Translational diffusion measured by PFG-NMR on full length and fragments of the Alzheimer Aβ (1–40) peptide. Determination of hydrodynamic radii of random coil peptides of varying length. Magn. Reson. Chem. 2002, 40, S89–S97. 10.1002/mrc.1132.

[ref98] Karlin D.; Longhi S.; Receveur V.; Canard B. The N-terminal domain of the phosphoprotein of morbilliviruses belongs to the natively unfolded class of proteins. Virology 2002, 296, 251–262. 10.1006/viro.2001.1296. 12069524

[ref99] Longhi S.; Receveur-Bréchot V.; Karlin D.; Johansson K.; Darbon H.; Bhella D.; Yeo R.; Finet S.; Canard B. Canard, B. The C-terminal domain of the measles virus nucleoprotein is intrinsically disordered and folds upon binding to the C-terminal moiety of the phosphoprotein. J. Biol. Chem. 2003, 278, 18638–18648. 10.1074/jbc.M300518200. 12621042

[ref100] Sánchez-Puig N.; Veprintsev D. B.; Fersht A. R. Human full-length Securin is a natively unfolded protein. Protein Sci. 2005, 14, 1410–1418. 10.1110/ps.051368005. 15929994 PMC2253381

[ref101] Sánchez-Puig N.; Veprintsev D. B.; Fersht A. R. Binding of natively unfolded HIF-1α ODD domain to p53. Mol. Cell 2005, 17, 11–21. 10.1016/j.molcel.2004.11.019. 15629713

[ref102] Khaymina S. S.; Kenney J. M.; Schroeter M. M.; Chalovich J. M. Fesselin is a natively unfolded protein. J. Proteome Res. 2007, 6, 3648–3654. 10.1021/pr070237v. 17676886

[ref103] Zhang X.; Perugini M. A.; Yao S.; Adda C. G.; Murphy V. J.; Low A.; Anders R. F.; Norton R. S. Solution conformation, backbone dynamics and lipid interactions of the intrinsically unstructured malaria surface protein MSP2. J. Mol. Biol. 2008, 379, 105–121. 10.1016/j.jmb.2008.03.039. 18440022 PMC4432223

[ref104] Mittag T.; Orlicky S.; Choy W.-Y.; Tang X.; Lin H.; Sicheri F.; Kay L. E.; Tyers M.; Forman-Kay J. D. Dynamic equilibrium engagement of a polyvalent ligand with a single-site receptor. Proc. Natl. Acad. Sci. U. S. A. 2008, 105, 17772–17777. 10.1073/pnas.0809222105. 19008353 PMC2582940

[ref105] Paz A.; Zeev-Ben-Mordehai T.; Lundqvist M.; Sherman E.; Mylonas E.; Weiner L.; Haran G.; Svergun D. I.; Mulder F. A.; Sussman J. L.; et al. Biophysical characterization of the unstructured cytoplasmic domain of the human neuronal adhesion protein neuroligin 3. Biophys. J. 2008, 95, 1928–1944. 10.1529/biophysj.107.126995. 18456828 PMC2483779

[ref106] Habchi J.; Mamelli L.; Darbon H.; Longhi S. Structural disorder within Henipavirus nucleoprotein and phosphoprotein: from predictions to experimental assessment. PLoS One 2010, 5, e11684 10.1371/journal.pone.0011684. 20657787 PMC2908138

[ref107] Choi U. B.; McCann J. J.; Weninger K. R.; Bowen M. E. Beyond the random coil: stochastic conformational switching in intrinsically disordered proteins. Structure 2011, 19, 566–576. 10.1016/j.str.2011.01.011. 21481779 PMC3075556

[ref108] Perez R. B.; Tischer A.; Auton M.; Whitten S. T. Alanine and proline content modulate global sensitivity to discrete perturbations in disordered proteins. Proteins: Struct., Funct., Bioinf. 2014, 82, 3373–3384. 10.1002/prot.24692. PMC4237723 25244701

[ref109] Yarawsky A. E.; English L. R.; Whitten S. T.; Herr A. B. The proline/glycine-rich region of the biofilm adhesion protein Aap forms an extended stalk that resists compaction. J. Mol. Biol. 2017, 429, 261–279. 10.1016/j.jmb.2016.11.017. 27890783 PMC5363081

[ref110] Poznar M.; Hołubowicz R.; Wojtas M.; Gapiński J.; Banachowicz E.; Patkowski A.; Ożyhar A.; Dobryszycki P. Structural properties of the intrinsically disordered, multiple calcium ion-binding otolith matrix macromolecule-64 (OMM-64). Biochimica et Biophysica Acta (BBA)-Proteins and Proteomics 2017, 1865, 1358–1371. 10.1016/j.bbapap.2017.08.019. 28866388

[ref111] Wiȩch A.; Rowińska-Żyrek M.; Wa̧tły J.; Czarnota A.; Hołubowicz R.; Szewczuk Z.; Ożyhar A.; Orłowski M. The intrinsically disordered C-terminal F domain of the ecdysteroid receptor from Aedes aegypti exhibits metal ion-binding ability. J. Steroid Biochem. Mol. Biol. 2019, 186, 42–55. 10.1016/j.jsbmb.2018.09.008. 30243841

[ref112] Marsh J. A.; Forman-Kay J. D. Sequence determinants of compaction in intrinsically disordered proteins. Biophys. J. 2010, 98, 2383–2390. 10.1016/j.bpj.2010.02.006. 20483348 PMC2872267

[ref113] Le Guillou J.; Zinn-Justin J. Critical exponents for the n-vector model in three dimensions from field theory. Phys. Rev. Lett. 1977, 39, 95 10.1103/PhysRevLett.39.95.

[ref114] Ding F.; Jha R. K.; Dokholyan N. V. Scaling behavior and structure of denatured proteins. Structure 2005, 13, 1047–1054. 10.1016/j.str.2005.04.009. 16004876

[ref115] Philo J. S. SEDNTERP: a calculation and database utility to aid interpretation of analytical ultracentrifugation and light scattering data. Eur. Biophys. J. 2023, 52, 233–266. 10.1007/s00249-023-01629-0. 36792822

[ref116] Wilkins D. K.; Grimshaw S. B.; Receveur V.; Dobson C. M.; Jones J. A.; Smith L. J. Hydrodynamic radii of native and denatured proteins measured by pulse field gradient NMR techniques. Biochemistry 1999, 38, 16424–16431. 10.1021/bi991765q. 10600103

[ref117] Ghafouri H.; Lazar T.; Del Conte A.; Tenorio Ku L. G.; Aspromonte M. C.; Bernadó P.; Chaves-Arquero B.; Chemes L. B.; Clementel D.; Cordeiro T. N.; et al. PED in 2024: improving the community deposition of structural ensembles for intrinsically disordered proteins. Nucleic Acids Res. 2024, 52, D536–D544. 10.1093/nar/gkad947. 37904608 PMC10767937

[ref118] Waszkiewicz R. glm-mda-diffusion. 2023. https://github.com/RadostW/glm-mda-diffusion (accessed 2023-12-25).

