# Supplementary material for: Hydrodynamic Radii of Intrinsically Disordered Proteins: Fast Prediction by Minimum Dissipation Approximation and Experimental Validation
Source: J Phys Chem Lett. 2024 May 2;15(19):5024–33. doi: 10.1021/acs.jpclett.4c00312 (PMC11103702; doi:10.1021/acs.jpclett.4c00312)
Supplement: Supplementary file 3 — jz4c00312_si_003.pdf [file jz4c00312_si_003.pdf]

jz-2024-00312u.R1

Name: Peer Review Information for "Hydrodynamic Radii of Intrinsically Disordered Proteins: Fast Prediction by Minimum Dissipation Approximation and Experimental Validation"

First Round of Reviewer Comments

Reviewer: 1

Comments to the Author

Report on *J Phys Chem Lett* manuscript jz-2024-00312u by Waszkiewicz *et al*.

This is a potentially interesting study offering a computationally efficient means to predict the hydrodynamic radii of intrinsically disordered proteins (IDPs) and proteins with both intrinsically disordered regions (IDRs) and folded domains. However, as it stands, the description of the authors' model is inadequate. As a result, it is not clear to the readers what assumptions are included or excluded in their chain model. Moreover, the discussion in the manuscript of related recent advances in the theoretical and computational studies of IDPs is insufficient, failing to address, for example, why information about amino acid sequences is excluded in the authors' model. To clarify the formulation and to put this work into a proper context, the following items need to be addressed before this manuscript can be reconsidered for publication.

1. There is no formal mathematical description of the authors' chain model in either the main text of supporting information. This is a conspicuous omission. A complete description with appropriate formulas must be provided. If length limitation in the main text is an issue, a brief description should be given in the main text but all the necessary equations for potential functions, etc. should be included in supporting information. Citing Github codes is useful but is insufficient by itself.

2. In particular, it is not clear whether the authors' model considers excluded volume or not. Van der Waals radii of various beads for individual amino acid residues and for folded domains are given so apparently excluded volume is included. But there is no explicit statement one way or the other. This feature of the model has to be clearly delineated.

3. It is not entirely why the authors do not consider the difference between different types of amino acids ("… Disordered segments of length *N* were modeled as chains of *N* identical spheres …" p.8 of the manuscript). If their model accounts for excluded volume (cf. point #2 above), the computation would already involve a consideration of pairwise excluded-volume interactions. On that basis, it should not be much more computationally intensive to include pairwise favorable or unfavorable interactions between different types of amino acid residues. It has been well

established that heteropolymers of the same length but different sequences can have very different conformational properties such as hydrodynamic radii (as showcased theoretically in ref.76 [Vovk & Zilman, 2023] cited in the manuscript and Lin & Chan, *Biophys J* **112:**2043-2046 (2017)). Therefore, not having the ability to account for sequence-dependent behavior is a critical deficiency of the authors' approach.

4. In the absence of an actual evaluation of the potential improvement on the authors' model by including sequence effects, the authors' assertion on p.14 of the manuscript that "Although explicit intramolecular interactions of the amino acid residues are neglected in MDA-GLM approach, the main cause of discrepancies between the experimental and predicted R_h values (Figure S8) appears to be the intrinsic properties of individual experimental methods, …" does not appear to be on solid ground. Would the authors make the same argument about experimental deficiencies if their predictions happen to agree better with the experimental data? Even if the authors were correct in pointing out that the experimental data were not entirely reliable, it is not clear how they would argue that the "corrected" data will fit better with their prediction because the proposed corrections can, *a priori*, lead to discrepancies with the predicted values in in a direction opposite to the original discrepancy. All in all, while the argument in the paragraph on p.14-p.15 is informative, it does not address not absolve the lack of consideration of sequence dependence, despite the connotation at the beginning of this paragraph.

5. At least two sets of recently optimized amino acid-dependent energies have been shown to be highly effective in capturing IDP conformational properties [Tesei et al., *Nature* **626:**897-904 (2024); Lotthammer et al., *Nature Methods* **21:**465-476 (2024)]. These sequence-specific interactions can readily be applied to at least the IDR parts of the proteins the authors considered. For the reasons discussed above in this referee report, the authors should either attempt to incorporate these energies in their model or provide a convincing explanation why they choose not to do so.

6. A key feature of IDPs that underpins their biological functions is their conformational heterogeneity (deviation from homopolymeric properties), entailing sequence-specific effects on conformational dimensions as characterized by end-to-end distances, radii of gyration R_g, hydrodynamic radii, etc., as has been investigated in the context of single-molecule FRET and SAXS experiments using examples such as Sic1 and Protein L [Song et al., *J Phys Chem B* **119:**15191-15202 (2015); Song et al., *J Phys Chem B* **125:**6451-6478 (2021)]. The perspective of these prior theoretical works should be included in the revised discussion, especially around the authors' introductory discussion on R_g and SAXS in the middle of p.4 of the manuscript.

A revised version of this work with all the points and related references listed above adequately cited and addressed can be reconsidered for publication in *J Phys Chem Lett*.

Author's Response to Peer Review Comments:

April 10th, 2024

Rebuttal letter to Reviewer's report

This is a potentially interesting study offering a computationally efficient means to predict the hydrodynamic radii of intrinsically disordered proteins (IDPs) and proteins with both intrinsically disordered regions (IDRs) and folded domains. However, as it stands, the description of the authors' model is inadequate. As a result, it is not clear to the readers what assumptions are included or excluded in their chain model. Moreover, the discussion in the manuscript of related recent advances in the theoretical and computational studies of IDPs is insufficient, failing to address, for example, why information about amino acid sequences is excluded in the authors' model. To clarify the formulation and to put this work into a proper context, the following items need to be addressed before this manuscript can be reconsidered for publication.

AUTHORS' REPLY: We are very grateful to the Reviewer for highlighting the shortcomings of the manuscript. According to His/Her advice, we have taken measures to rectify these issues by expanding upon deficient sections within the main text and providing additional commentary in the Supporting Information. Detailed responses to each concern and the description of the introduced amendments and changes are outlined below. Concurrently, we attach the manuscript file with the tracked changes as "Supporting Information for Review Only".

1. There is no formal mathematical description of the authors' chain model in either the main text or supporting information. This is a conspicuous omission. A complete description with appropriate formulas must be provided. If length limitation in the main text is an issue, a brief description should be given in the main text but all the necessary equations for potential functions, etc. should be included in supporting information. Citing Github codes is useful but is insufficient by itself.

2. In particular, it is not clear whether the authors' model considers excluded volume or not. Van der Waals radii of various beads for individual amino acid residues and for folded domains are given so apparently excluded volume is included. But there is no explicit statement one way or the other. This feature of the model has to be clearly delineated.

AUTHORS' REPLY: We concur with the Reviewer's assessment that the description of the conformer generation scheme lacked some detail and required further elaboration for the complete construction of appropriate distributions. To address this concern, we have expanded the section of the Main Text (pages 8 to 10) detailing the self-avoiding random walk of spheres. In response to the reviewer's feedback, we have also opted to separate the mathematical description of the sampled distribution from the sampling algorithm itself. This separation allows for a more detailed explanation of the sampling algorithm in the Supporting Information, which now includes condensed pseudocode for precise understanding without delving into implementation specifics. We hope that in the current manuscript form, we have provided a complete and comprehensive description of the conformer generation method.

Additionally, we have improved this section of the Main Text by explicitly mentioning the inclusion of excluded volume interactions (page 10), which are indeed central to the model's

conceptual framework. This statement about exclusion volume effects is also underlined in the concluding remarks (last sentence of the text, page 17).

3. It is not entirely clear why the authors do not consider the difference between different types of amino acids ("… Disordered segments of length N were modeled as chains of N identical spheres …" p.8 of the manuscript). If their model accounts for excluded volume (cf. point #2 above), the computation would already involve a consideration of pairwise excluded-volume interactions. On that basis, it should not be much more computationally intensive to include pairwise favorable or unfavorable interactions between different types of amino acid residues. It has been well established that heteropolymers of the same length but different sequences can have very different conformational properties such as hydrodynamic radii (as showcased theoretically in ref.76 [Vovk & Zilman, 2023] cited in the manuscript and Lin & Chan, Biophys J 112:2043-2046 (2017)). Therefore, not having the ability to account for sequence-dependent behavior is a critical deficiency of the authors' approach.

AUTHORS' REPLY: We would like to thank the Reviewer for turning our attention to the need for a clarification of the issue on how our model relates to the sequence-dependent properties of IDPs. The question of which properties of Intrinsically Disordered Proteins (IDPs) should be included in hydrodynamic models can also be examined from another perspective: Which features of IDPs can be effectively studied through hydrodynamic measurements? We agree with the Reviewer that the specifics of the sequence are crucial and have a significant impact on both the conformational ensemble and function of IDPs. This aligns with our model, which suggests that the influence of the sequence is mediated through the positioning of the boundaries of the protein's globular fragments. The sequence specificity is thus implicitly involved in our IDP model in the range of domains, by using two semi-empirical constraints, i.e. the boundaries of ordered fragments derived from Dispored3 and the length of loops allowed between the secondary structural elements that form together a singular globular domain. This is now clarified explicitly on pages 8-9.

One of the findings of our work is that the sizes and positions of the globular domains within the intrinsically disordered protein chain proved to be the dominating factors that influence the hydrodynamic properties of the IDP chain as a whole. In the revised version of the text, this is discussed more clearly on page 15 and summarized on page 16.

According to the Reviewer's advice, in the revised version of the manuscript, the question of how the charge patterning of the sequence can affect the IDP dimensions has also been addressed by reference to Lin & Chan, Biophys J 112:2043-2046 (2017) on page 9. Their work, and similarly the work by Vovk & Zilman, 2023, focuses on polyampholytic sequences that do not contain any globular domains. In such a case, the Coulombic interactions can play a major role. In particular, the work of Vovk and Zilman compares sensitivity of several features of IDPs to the details of the sequence (cf. Figure 1 therein) with hydrodynamic size being the least sensitive of the outcome variables studied. Moreover, the effect of "sequence charge decoration" for hydrodynamic size (cf. Figure 4 ibid) in the most extreme case (the first half of the chain is composed of all positively charged while the second is all negatively charged residues) is of the order of 40% when simulated in a buffer of ionic strength lower than in our investigation, for a protein of length N=50 (much shorter than many of proteins in our investigation), and when the hydrodynamic radius is estimated using the Kirkwood approximation. This value has to be treated as an upper bound to the size of possible effects

in experiments. We agree that the electrostatic interactions discussed by e.g. Lin & Chan, Biophys J 112: 2043-2046 (2017), Das, R. K. et al., Current Opin Struct Biol (2015), Vovk & Zilman (2023) are present and influence the radius of gyration, hydrodynamic radius and conformation in general; these effects should be included in future refinements of our work, as we mentioned in the last paragraph of the text on page 17.

These effects are, however, of secondary importance when compared with strong, direct, short-range interactions of the hydrophobic residue side chains that lead to the formation of globular fragments of the protein. These are accounted for by the Disopred3 predictions used, and mediated by the location of the globule-linker boundaries (pages 8-9). Proper accounting for effectively rigid domains is vital for the good prediction of the hydrodynamic size, since they interact both directly via excluded volume interactions and indirectly by entropic repulsion of the linkers from the domains (due to excluded volume interaction within those).

The excluded volume interactions can be expressed as a repeated conditioning of the distribution, allowing for the application of the recursive algorithm for conformer generation which would not be possible when electrostatic interactions are added. The inclusion of sequence-specific effects is not simply the case of changing a potential in our sampling algorithm. We would have to switch the sampling method to a Monte Carlo simulation, which would significantly increase the computational burden and slow down the procedure, which is contrary to the scientific goal of this manuscript. Our aim was to create and validate a speedy and efficient method to get Rh values for every IDP without a length constraint, that is sufficiently close to the expected experimental value with an accuracy comparable to experimental uncertainty, without requiring extensive numerical simulations. The results presented in Fig. 4, Table 1, and Fig. S1 suggest that we are indeed close to achieving this goal.

4. In the absence of an actual evaluation of the potential improvement on the authors' model by including sequence effects, the authors' assertion on p.14 of the manuscript that "Although explicit intramolecular interactions of the amino acid residues are neglected in MDA-GLM approach, the main cause of discrepancies between the experimental and predicted Rh values (Figure S8) appears to be the intrinsic properties of individual experimental methods, …" does not appear to be on solid ground. Would the authors make the same argument about experimental deficiencies if their predictions happen to agree better with the experimental data? Even if the authors were correct in pointing out that the experimental data were not entirely reliable, it is not clear how they would argue that the "corrected" data will fit better with their prediction because the proposed corrections can, a priori, lead to discrepancies with the predicted values in a direction opposite to the original discrepancy. All in all, while the argument in the paragraph on p.14-p.15 is informative, it does not address not absolve the lack of consideration of sequence dependence, despite the connotation at the beginning of this paragraph.

AUTHORS' REPLY: We agree with the Reviewer that the formulation of the sentence "Although explicit intramolecular interactions of the amino acid residues are neglected in MDA-GLM approach, the main cause of discrepancies between the experimental and predicted Rh values (Figure S8) appears to be the intrinsic properties of individual experimental methods, …" could be misleading. In fact, intramolecular interactions of the

amino acid residues are not neglected in the MDA-GLM approach, but included implicitly in the ranges of folded domain sequences by using semi-empirical Disopred3 data, together with the empirical upper limit of loop length allowed within a singular domain. The sequence specificity effects are neglected only for the linker fragments, which is indeed one of the possible sources of uncertainty linked to our model. However, in our opinion, it is an acceptable level of error for such a quick numerical method. According to the Reviewer's remarks, this issue is now discussed more profoundly by adding a better model description on pages 8-9, an additional paragraph of discussion on page 15, and a comment regarding the limitation of the model in the concluding remarks on page 17.

Moreover, our former statement could lead to the conclusion that experimental methodological issues were the only cause of the disagreement between the theoretical predictions and the experimental results. Following the Reviewer's comments, we have changed the text on page 15 regarding the experimental contribution to the discrepancies. Still, one should bear in mind that such a theory vs. experiment comparison requires an additional commentary for the reader, which would indicate and explain the limitations and differences among experimental methods by means of which the hydrodynamic parameters are measured in a more or less direct way. For this reason, a discussion on the most likely experimental sources of uncertainty was included in the manuscript together with a set of supplementary figures in the Supporting Information (Fig. S8) to better illustrate the arguments described on pages 15-16.

Nevertheless, the assessment of the accuracy of our method using six metrics (pages 13-14, Table1) showed that even neglecting the effects resulting from the specificity of the linker sequence, for the widest set of IDPs, we obtained more accurate results than any previously used coarse-grained or phenomenological prediction methods.

5. At least two sets of recently optimized amino acid-dependent energies have been shown to be highly effective in capturing IDP conformational properties [Tesei et al., Nature 626:897-904 (2024); Lotthammer et al., Nature Methods 21:465-476 (2024)]. These sequence-specific interactions can readily be applied to at least the IDR parts of the proteins the authors considered. For the reasons discussed above in this referee report, the authors should either attempt to incorporate these energies in their model or provide a convincing explanation why they choose not to do so.

AUTHORS' REPLY: We agree with the Reviewer that the achievements of the groups of Lindorff-Larsen and Holehouse should be acknowledged. In the amended version of the manuscript, we refer to their works in the introductory discussion on page 4, as well as in the last, concluding paragraph of the text on page 17. The issue related to the application of the above-mentioned sequence-specific interactions is already explained by us in section 3 of this rebuttal letter. Incorporation of the explicit sequence-dependent energies would preclude the achievement of the main objective of the manuscript, which is to show that it is possible to rapidly predict Rh values of IDPs within the limits of experimental uncertainty by applying the Minimum Dissipation Approximation and the Globule-Linker Model.

It is worth mentioning that there are examples known from literature (e.g. Song J. et al. J Phys Chem B, 2015, 119, 15191–15202, cited as ref. no. 33 in the corrected manuscript) where an accurate Rh value of an intrinsically disordered Sic1 fragment could be obtained

by using a protein chain model that was composed of indistinguishable beads, providing that the excluded volume effects were precisely elaborated. This is now included in the introductory part on page 4.

6. A key feature of IDPs that underpins their biological functions is their conformational heterogeneity (deviation from homopolymeric properties), entailing sequence-specific effects on conformational dimensions as characterized by end-to-end distances, radii of gyration Rg, hydrodynamic radii, etc., as has been investigated in the context of single-molecule FRET and SAXS experiments using examples such as Sic1 and Protein L [Song et al., J Phys Chem B 119:15191-15202 (2015); Song et al., J Phys Chem B 125:6451-6478 (2021)]. The perspective of these prior theoretical works should be included in the revised discussion, especially around the authors' introductory discussion on Rg and SAXS in the middle of p.4 of the manuscript.

AUTHORS' REPLY: According to the Reviewer's valuable suggestions, we have complemented our introductory part on page 4 by adding a discussion regarding the pitfalls on the way of inferring structural properties of the IDP conformational ensembles from SAXS. This issue was previously exhaustively elaborated and mathematically proven by Song et al., J Phys Chem B 125:6451-6478 (2021) (ref. 32 in the corrected manuscript). We have also added an example that revealed the critical importance of proper modeling of the excluded volume effect for estimating the Rh value of the disordered N-terminal Sic1 fragment, which was previously shown by Song et al., J Phys Chem B 119:15191-15202 (2015) (ref. 33). Accordingly, we have also cited the original source of the experimental data (Liu, B. et al. J Phys Chem B 2014, 118, 4088–4097, ref. 34).

A revised version of this work with all the points and related references listed above adequately cited and addressed can be reconsidered for publication in J Phys Chem Lett.

AUTHORS' REPLY: We would like to express our greatest gratitude to the Reviewer for suggesting amendments to our paper. We hope that all the issues raised in the review have been successfully resolved, ambiguities have been removed and clarifications have been made, as requested.

We would also like to mention that in the course of the amendments, we have added to Table 1 and Figure 4 the results obtained from another phenomenological approach developed to account for the absolute net charge in addition to the polyproline II propensities of the protein sequence (English LR, et al., Proteins. 2017, 85(2): 296-311, cited as ref. no. 31).

Sincerely,

Piotr Szymczak, PhD & Anna Niedźwiecka, PhD
